# Low-dose arecoline regulates distinct core signaling pathways in oral submucous fibrosis and oral squamous cell carcinoma

**DOI:** 10.1186/s12903-023-02887-2

**Published:** 2023-03-25

**Authors:** Zhenming Li, You Fu, Yuhua Hu, Yun Zhu, Longwei Hu, Chaoji Shi, Yi Zhang, Jianjun Zhang, Shanghui Zhou

**Affiliations:** 1 Department of Oral & Maxillofacial - Head & Neck Oncology, Shanghai Ninth People’s Hospital, Shanghai Jiao Tong University School of Medicine, Shanghai, 200011 China; 2 College of Stomatology, Shanghai Jiao Tong University, Shanghai, 200011 China; 3 National Center for Stomatology, Shanghai, 200011 China; 4 National Clinical Research Center for Oral Diseases, Shanghai, 200011 China; 5 Shanghai Key Laboratory of Stomatology, Shanghai, 200011 China; 6 Shanghai Research Institute of Stomatology, Shanghai, 200011 China; 7 Department of Oral Pathology, Shanghai Ninth People’s Hospital, Shanghai Jiao Tong University School of Medicine, Shanghai, 200011 China; 8 Department of Endodontics and Operative Dentistry, Shanghai Ninth People’s Hospital, Shanghai Jiao Tong University School of Medicine, Shanghai, 200011 China

**Keywords:** Arecoline, OPMD, OSF, OSCC, Malignant progression

## Abstract

**Background:**

Betel nut chewing plays a role in the pathogenesis of oral submucous fibrosis (OSF) and oral squamous cell carcinoma (OSCC). As the major active ingredient of the betel nut, the effect of arecoline and its underlying mechanism to OSF and OSCC pathogenesis remain unclear.

**Methods:**

Next-generation sequencing-based transcriptome and dRRBS analysis were performed on OSF and OSCC cells under low-dose arecoline exposure. Functional analyses were performed to compare the different roles of arecoline during OSF and OSCC pathogenesis, and key genes were identified.

**Results:**

In this study, we identified that low-dose arecoline promoted cell proliferation of both NFs and OSCC cells via the acceleration of cell cycle progression, while high-dose arecoline was cytotoxic to both NFs and OSCC cells. We performed for the first time the transcriptome and methylome landscapes of NFs and OSCC cells under low-dose arecoline exposure. We found distinct transcriptome and methylome profiles mediated by low-dose arecoline in OSF and OSCC cells, as well as specific genes and signaling pathways associated with metabolic disorders induced by low-dose arecoline exposure. Additionally, low-dose arecoline displayed different functions at different stages, participating in the modulation of the extracellular matrix via Wnt signaling in NFs and epigenetic regulation in OSCC cells. After exposure to low-dose arecoline, the node roles of *FMOD* in NFs and histone gene clusters in OSCC cells were found. Meanwhile, some key methylated genes induced by arecoline were also identified, like *PTPRM* and *FOXD3* in NFs, *SALL3* and *IRF8* in OSCC cells, indicating early molecular events mediated by arecoline during OSF and OSCC pathogenesis.

**Conclusions:**

This study elucidated the contribution of low-dose arecoline to OSF and OSCC pathogenesis and identified key molecular events that could be targeted for further functional studies and their potential as biomarkers.

**Supplementary Information:**

The online version contains supplementary material available at 10.1186/s12903-023-02887-2.

## Background

Betel nut chewing is a common habit in Asian areas including India, Taiwan and mainland China [1]. In recent years, chewing betel nut has also become prevalent in Western countries such as the United Kingdom, the United States, and other developed countries [2]. Approximately 10–20% of the population worldwide chew some form of betel nut regularly, thus betel nut now has become the fourth most consumed psychoactive substance after nicotine, ethanol and caffeine [3]. World Health Organization classified betel nut as a Group I carcinogen. Arecoline is the primary biologically active alkaloid of betel nuts [4].

Chewing betel nuts is closely associated with the development of oral potentially malignant disorders (OPMD) and oral cancer, like oral submucous fibrosis (OSF), oral leukoplakia when combined with tobacco, and oral squamous cell carcinoma (OSCC). OSF malignant transformation has increased in recent years, reaching 3%-19% as a premalignant lesion [5]. Oral squamous cell carcinoma in the background of/with oral submucous fibrosis (OSCC-OSF) developed from betel nut chewing has been one of the common malignancies in areas with the habit of chewing betel nut [6].

OSCC-OSF developed from betel nut chewing presents subtle differences from ordinary OSCC clinically [7]. Also, OSCC-OSF has unique manifestations in clinical pathology, such as better grades of tumor differentiation, less incidence of nodal metastases, and less extracapsular spread (ECS) [6, 7]. The presence of OSF is an independent predictive factor for OSCC lymph node metastasis. Fibrosis is the unique feature of OSF, unlike other oral premalignant disorders. As an important environmental risk factor for OSF and oral cancer, the molecular mechanisms of arecoline induction contributing to OSF and OSCC pathogenesis remain still unclear.

Epigenetic changes are considered as the earliest and most comprehensive genomic aberrations occurring during tumor pathogenesis, facilitating tumor initiation and progression [8]. Single gene-based studies have shown that arecoline-induced methylation dysregulation of a few genes including *DUSP4* [9], *RARB* [10] and *SIRT1* [11]. We previously identified Wnt antagonist genes hypermethylation (*WIF1, SFRPs*) in OSF and OSCC-OSF tissues [12, 13], suggesting the importance of epigenetic alterations during arecoline-induced OSF and OSCC-OSF pathogenesis. Despite intensive applications of next-generation sequencing in OSCC study, genome-wide transcriptome and methylome profiling of arecoline-induced alterations during OSF and OSCC pathogenesis have not been conducted.

In this study, we performed transcriptome and methylome landscapes of normal fibroblasts from oral mucosal (NFs) and OSCC cells under low-dose arecoline exposure using RNA-sequencing and dRRBS platforms. Collectively, these findings delineated that epigenetic reprogramming-mediated transcriptome changes play important roles in the occurrence and development of arecoline-induced OSF and OSCC progression, and identified some key molecular events involved in metabolic disorders and epigenetic regulation, which will help develop new potential biomarkers and therapeutic targets for the treatment of OSF and OSCC caused by chewing areca nuts.

## Methods

### Cell lines

Human tongue squamous cells (CAL27) and human primary buccal mucosal fibroblasts (NFs) were obtained from the Shanghai Key Laboratory of Stomatology. Both cells were cultured under the same conditions, containing DMEM medium with 10% fetal bovine serum, and supplemented with 1% penicillin‐streptomycin in a humidified 5% CO2 incubator at 37 °C.

### Cell counting kit-8 assay

Arecoline (Absin Bioscience Inc) was dissolved in phosphate-buffered saline (PBS) at different concentrations of 640, 320, 160, 80, 40, 20, 3.125, 0.625, 0.125, 0.025, and 0.005 μg/ml respectively. CAL27 cells and NFs (5 × 10^3^ per well) were seeded in 96-well culture plates and all experimental groups were incubated for 24 h. The high-concentration groups were inducted by arecoline at 0, 20, 40, 80, 160, 320, and 640 μg/ml respectively and the low-concentration groups were inducted by arecoline at 0, 3.125, 0.625, 0.125, 0.025, and 0.005 μg/ml respectively. Cell Counting Kit-8 (CCK8, Dojindo, Japan) was used to assess cell proliferation. CCK-8 reagent was added after 24 h induction and then the absorbance was measured.

The proliferation of CAL27 cells and NFs was analyzed with the following formula: %cell inhibition rate = 100 − (mean optical density (OD) of the treatment wells − mean OD of the background control wells)/(mean OD of the negative control wells – mean OD of the background control wells) × 100.

### Flow cytometry

Arecoline-treated CAL27 cells were first starved without serum for 24 h. The culture medium was then modified with 1.25 μg/ml arecoline in CAL27 cells and 0.625 μg/ml arecoline in NFs for 24 h. After trypsinization, cells were digested with 0.25% trypsin and washed twice with cold PBS, and then fixed with 70% ethanol for 24 h at 4℃. The cells were incubated for 30 min by freshly prepared propidium iodide (PI) solution containing 1 × binding buffer, 20 μg/ml PI (BD Pharmingen, Heidelberg, Germany), and 0.5U RNase A (Sigma, USA) and assessed by flow cytometry. Flowjo software (Becton, Dickinson & Company, USA) was used to analyze the data.

### DNA methylome profiling of low-dose arecoline-induced NFs and CAL27 cells

Firstly, NFs and CAL27 cells were cultured with 0.625 μg/ml and 1.25 μg/ml arecoline for 24 h respectively. The CAL27 cell experimental group with arecoline treatment (C1E), CAL27 cell control group (C1C), NF experimental group with arecoline treatment  (NF1E), and NF control group (NF1C) were set up. Genomic DNA was digested using the MspI enzyme for 16 h at 37 °C. After digestion, libraries were constructed as the Illumina pair-end protocol. A mix of T4 DNA polymerase, Klenow fragment, and T4 polynucleotide kinase was added. Next, the mixture was 3’ adenylated using the Klenow Fragment (3’-5’ exo-) and followed by ligation to adaptors synthesized with 5’-methylcytosine. The DNA was purified using the QIAquick PCR purification kit (Qiagen) after the reaction of each step. Before the oxidation reaction, all products had been purified by the TrueMethyl Seq Kit (CEGX) according to the manufacturer’s instructions. The oxidation reaction was conducted by the Oxidant Solution (CEGX), and the dRRBS library sample was added in 1ul of ultrapure water instead as the control. Both two libraries were subjected to 40 °C for 30 min treatment in a thermocycler with the lid heated at 57 °C. After that, centrifuged the reaction mixture and then transferred the supernatant into a new PCR tube for further bisulfite treatment, respectively. Bisulfite conversion treatment was performed using a TrueMethyl Seq Kit (CEGX) according to the manufacturer’s instructions. The final dRRBS libraries were generated by PCR amplification using adapter-compatible barcode primers, quantified by an Agilent 2100 Bioanalyzer (Agilent Technologies) and real-time PCR assay, and then sequenced by Illumina Hiseq.

The genome sequence was then divided into different gene structural elements according to the transcription initiation site, including 2000 bp upstream (up2k), 5’ terminal non-coding region (5’UTR), exon (exon), intron (intron), 3’ non-coding region (3’UTR), and downstream 2000 bp (down2k). The CG sites of up2k, 5’UTR, exon, intron, 3’UTR, and down2k in each transcript were counted with their methylation rate. Similarly, we verified the methylation level of CpG island (CGI). The CGI was determined according to the following criteria: 1. The CG content in the section >  = 50%; 2. Section length >  = 200 bp; 3. the ratio of observed to expected CpG dinucleotides in the segment (Obs/Exp CpG) >  = 0.6, where Obs/Exp CpG is calculated as follows: Obs/Exp CpG = Number of CpG * N / (Number of C * Number of G); N: The length of the gene segment.

Differentially Methylated Region (DMR) was detected by metilene using a binary segmentation algorithm combined with dual statistical tests (MWU-test and 2D KS-test). Finally, the DMR was obtained by multiple test corrections, and CpG sites were used to look for differential methylation regions. The DMR was detected by the rules below: (1) Sequencing depth per CpG site >  = 5x; (2) Methylation difference at CpG sites >  = 0.1; (3) Differential methylation of the region The number of CpG sites >  = 5; (4) Distance <  = 300 bp for adjacent differential methylation CpG sites; (5) MWU-test q-value < 0.05.

### Transcriptome analysis of low-dose arecoline-induced CAL27 cells and NFs

CAL27 cells and NFs were cultured under the same conditions as mentioned above, and the CAL27 cell experimental group (C1E), CAL27 cell control group (C1C), NF experimental group (NF1E), and NF control group (NF1C) were set up. RNA-seq detection was performed three times per group.

Total RNA was extracted from CAL27 cells and NFs treated with or without arecoline using a standard TRIzol extraction protocol. mRNA was enriched with magnetic beads with Oligo (dT). A fragmentation buffer was then added to break the mRNA into fragments and synthesize a chain of cDNA with six base random primers. Then dNTPs and DNA polymerase were added to synthesize double-stranded cDNA. The double-stranded DNA was purified using the amPure XP system. Next, PCR was performed, the products were purified (AMPure XP system), and the library quality was assessed using the Agilent Bioanalyzer 2100 system. After the insert size met expectations, the library was accurately quantified by the qPCR method (the effective concentration of the library > 2 nM) to ensure the quality of the library. The different libraries were pooled on the HiSeq platform according to the effective concentration and the target data volume requirements, and the sequencing strategy was PE150.

To identify the differentially expressed genes (DEGs),the gene expression was estimated by mapping the count of sequences (reads) to the genome region or the exon region of the gene. HTSeq software was used to analyze the gene expression level of each sample using the union model. Differential expression was performed between each of the two groups of samples using deseq, and genes with a *p*-value of less than 0.05 were selected as DEGs.

### Protein–protein interaction (PPI) network

To construct the protein–protein interaction (PPI) network between DEGs of NFs and CAL27 cells, the Search Tool for the Retrieval of Interacting Genes/ Proteins (STRING, https://string-db.org/) database was used. And STRING (version 11.0) was used to identify the interactions among the DEGs of NFs and CAL27 cells separately. The Cytoscape plugin Molecular Complex Detection (MCODE) was used to identify the top two modules in the PPI network using the following parameters: degree cutoff = 2, node density cutoff = 0.2, node score cutoff = 0.1, K-core = 2, and max. depth = 100. In addition, the cytoHubba plugin was used to calculate the degree of each protein node.

### GO and KEGG analysis of DEGs and differential methylation genes (DMGs)

To determine the most important biochemical metabolic pathway and signal transduction pathway that DEGs or DMGs participated in, GO and KEGG pathway analysis was performed. The GO database provides information on the functions of genes, including biological processes (BPs), cellular components (CCs), and molecular functions (MFs). KEGG is a web database for exploring advanced functions of biological systems (https://www.kegg.jp). GO annotated information is downloaded from Ensembl's Biomart database (http://asia.ensembl.org). The overlapping DEGs were submitted to GO and KEGG pathway analyses by The Database for Annotation, Visualization and Integrated Discovery (DAVID, https://david.ncifcrf.gov) tool (version 6.8). And the hypergeometric test was used to find out which Pathway was significantly enriched in differentially expressed genes compared with the whole genome background. GO enrichment analysis was conducted using topGO, and ClusterProfiler was used for Pathway enrichment analysis. A *P*-value of < 0.05 was considered statistically significant.

### Statistical analysis

GraphPad Prism software (version 8.0.0) was used for all statistical analysis and figure generation. *, **, ***, and **** were expressed as significant differences in *P* < 0.05, 0.01, 0.001, and 0.0001 respectively. To evaluate the changes in the cell cycle after the induction of arecoline, matched samples t-test was performed. Cell viability results of arecoline-treated and untreated cells were analyzed using one-way analysis of variance analysis (ANOVA) and Tukey tests, and a *P*-value of < 0.05 was considered to indicate a significant difference. 

## Results

### Unveiling the impact of different doses of arecoline exposure on cell growth in NFs and OSCC cells

**Fig. 1:**
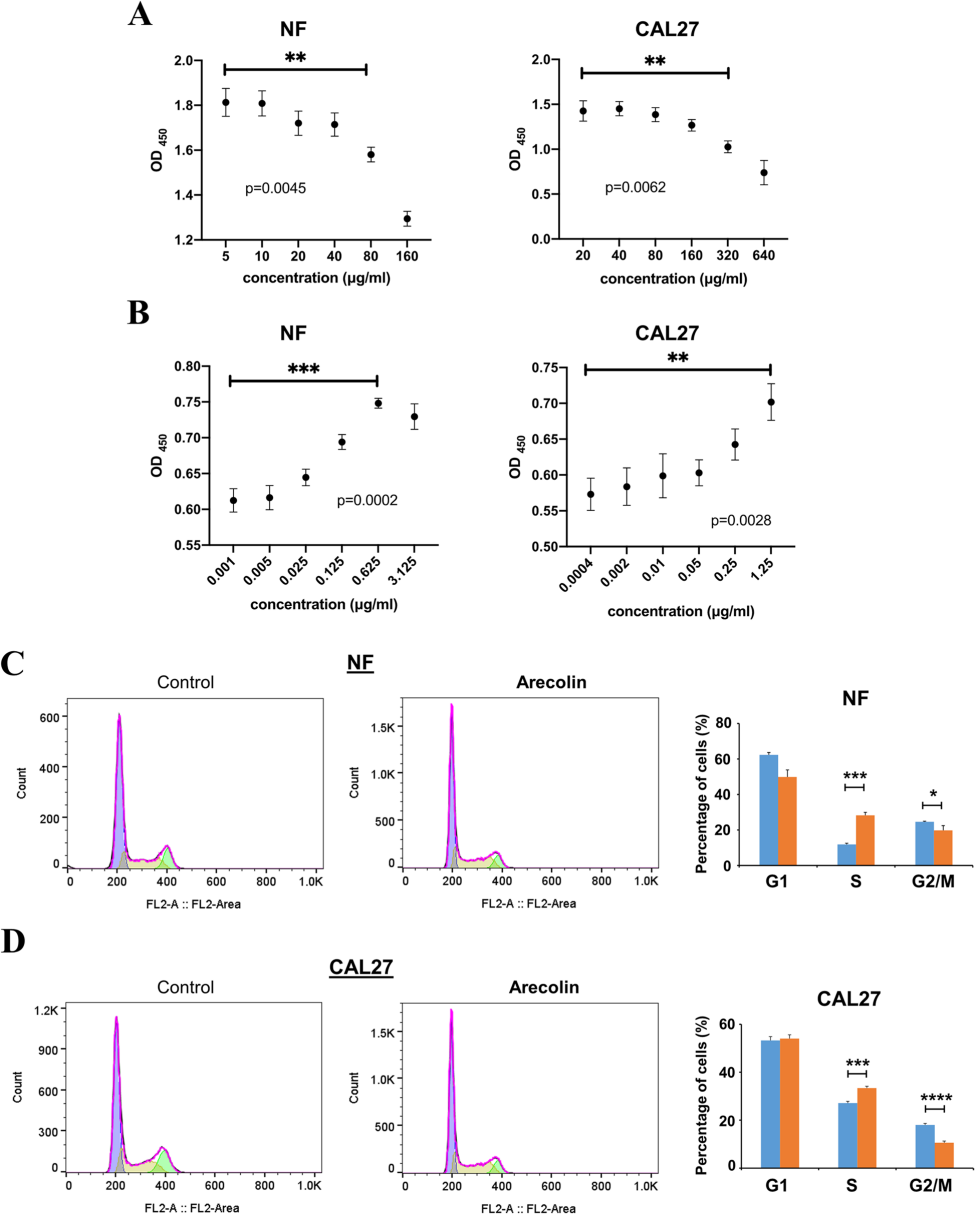
High-dose arecoline is cytotoxic to NFs and CAL27 cells but low-dose arecoline promotes the proliferation of NFs and CAL27 cells. **A**-**B** CCK-8 assay showing the viability of NFs and CAL27 cells after exposure to various concentrations of arecoline from 0.0004 μg/ml to 640 μg/ml for 24 h. Growth inhibition was detected in triplicate samples, and significant differences compared with control were noted by asterisks. ** *p* < 0.01; *** *p* < 0.001. **C**-**D** Effects of low-dose arecoline on cell cycle progression of NFs and CAL27 cells. Arecoline was treated NFs at 0.625 μg/ml (C) and CAL27 cells at 1.25 μg/ml (D) for 24 h. Changes in the percentage of cells in the G0/G1, S and G2/M phase in NFs and CAL27 cells with or without arecoline treatment were examined. Results were expressed as a percentage of cells in different phases (mean ± SE). Asterisks represented a significant difference when compared with controls. * *p* < 0.05, *** *p* < 0.001, **** *p* < 0.0001

Studies have shown that high doses of arecoline could inhibit human oral fibroblast proliferation, while low doses of arecoline could promote the proliferation of human oral fibroblasts. We first separated low-dose from high-dose effects of arecoline on cell growth in both human primary buccal mucosal fibroblasts (NF) and OSCC (CAL27) cells by CCK-8 assay. Different doses of arecoline in the high dose range were treated NF cells (5, 10, 20, 40, 80, or 160 μg/ml) and CAL27 cells (20, 40, 80, 160, 320, or 640 μg/ml) for 24 h, respectively. Results showed that cell viabilities of both NFs and CAL27 cells were decreased when the concentration of arecoline increased at a high dose range, and the obvious inhibitory effect on cell proliferation was observed at over 80 μg/ml in NF cells and over 320 μg/ml in CAL27 cells (Fig. 1A).

To mimic the oral microenvironment during betel nut chewing, we next evaluated the cell viability at low doses of arecoline in both NFs and CAL27 cells. Different doses of arecoline in the very low dose range were treated in NFs (ranging from 0.001—3.125 μg/ml) and CAL27 cells (ranging from 0.0004—1.25 μg/ml). Results showed that low-dose arecoline promoted cell proliferation of both NFs and CAL27 cells in a dose-dependent manner, with the maximum effect on proliferation observed at 0.625 μg/ml for NFs and 1.25 μg/ml for CAL27 cells (Fig. 1B).

To investigate the underlying mechanisms of low-dose arecoline on cell proliferation, the effect of cell cycle changes of NFs and OSCC cells was examined by flow cytometry. Exposure of NFs and CAL27 cells to arecoline led to significantly increased S phase cells and decreased G2/M phase cells, indicating that low doses of arecoline promoted the G1/S phase transition of NFs and CAL27 cells (Fig. 1C, D). These data suggest that high doses of arecoline inhibit the proliferation of NFs and OSCC cells, while low doses of arecoline promote both NFs and OSCC cell proliferation via acceleration of the G1/S phase transition during OSF and OSCC pathogenesis.

### Transcriptomic changes induced by the low-dose arecoline in NFs and OSCC cells

**Fig. 2:**
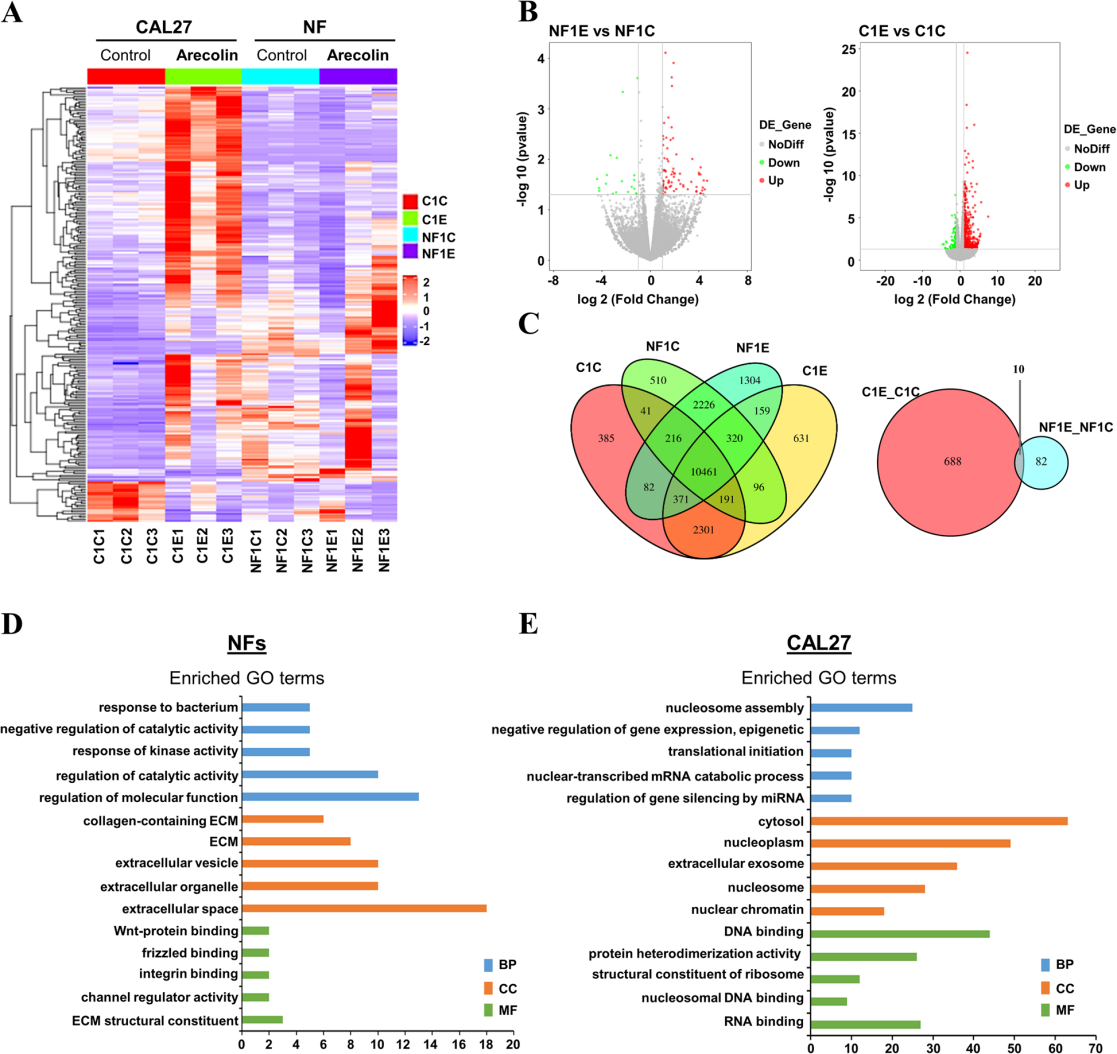
Low-dose arecoline effects on the transcriptome of NFs and CAL27 cells. **A** Hierarchical clustering analysis of transcriptome-wide gene expression patterns of DEGs in NFs and CAL27 cells under low-dose arecoline treatment. **B** Volcano plot showing gene expression profiles in DEGs of the low-dose arecoline group compared to the control group in NFs and CAL27 cells. Red and green spots represent upregulated and downregulated DEGs, respectively, and grey spots represent no different DEGs. **C** Venn diagram showing the genes expression (left) in NFs and CAL27 cells with or without low-dose arecoline treatment, as well as DEGs of NFs and CAL27 under low-dose arecoline treatment (right). **D**-**E** GO enrichment analysis of DEGs in low-dose arecoline-treated NFs and CAL27 cells. GO enrichment of DEGs of NFs and CAL27 cells under low-dose arecoline treatment in biological process (BP), molecular function (MF), and cell composition (CC). The Y-axis represents the different GO categories, and the X-axis represents the number of genes with significance in the GO category. *p*-value < 0.05 and/or q-value < 0.05 are considered statistically significant. GO, gene ontology; C1E, CAL27 cells with low-dose arecoline treatment; C1C, CAL27 cells without low-dose arecoline treatment; NF1E, NFs with low-dose arecoline treatment; NF1C, NFs without low-dose arecoline treatment

To investigate underlying mechanisms mediated by the low-dose arecoline during OSF and OSCC pathogenesis, transcriptomic changes upon low-dose arecoline exposure by RNA sequencing were performed in NFs and CAL27 cells after being treated with arecoline at 0.625 μg/ml and 1.25 μg/ml for 24 h respectively. To visualize differences between groups with or without arecoline treatment, a bootstrap hierarchical clustering was conducted and clearly showed differentially expressed gene (DEG) patterns in both NFs and CAL27 cells after exposure to low-dose arecoline (Fig. 2A). Moreover, in low-dose arecoline-treated groups, NFs and CAL27 cells also showed differences in gene expression profiles after treatment, indicating that low-dose arecoline plays different roles in OSF and OSCC pathogenesis.

Volcano plots revealed 92 genes differentially expressed after low-dose arecoline treatment in NFs including 73 upregulated genes and 19 downregulated genes, while 698 DEGs were found in CAL27 cells after low-dose arecoline exposure including 585 upregulated genes and 113 downregulated genes (Fig. 2B, Suppl. Table 1, Suppl. Table 2). Venn diagram analysis further revealed coexpressed genes among the four groups and found 10 common DEGs in both NFs and CAL27 cells after being treated with arecoline (Fig. 2C, Suppl. Table 3), including *cyclin-dependent kinase-like 1 (CDKL1)*, *mitochondrial ribosomal protein L12* (*MRPL12*), and ribosomal RNAs (*RNA5S9, RN7SL4P*), suggesting disruption of cell cycle and ribosomes induced by low-dose arecoline during OSF and OSCC pathogenesis.

### Functional signaling pathways affected by low-dose arecoline in NFs and OSCC cells

We then analyzed the potential functional roles of DEGs induced by low-dose arecoline in NFs and CAL27 cells. GeneOntology (GO) enrichment analysis of DEGs in NFs after low-dose arecoline exposure revealed significant enrichments in regulating catalytic activity, kinase activity, and oral bacteria, as well as binding to integrin and Wnt-protein, which led to extracellular matrix (ECM) remodelling (Fig. 2D, Suppl. Table 4). In CAL27 cells under low-dose arecoline treatment, DEGs were involved in nucleosome assembly and epigenetic regulation of gene expression via DNA/RNA and protein dimerization (Fig. 2E, Suppl. Table 5).

**Fig. 3:**
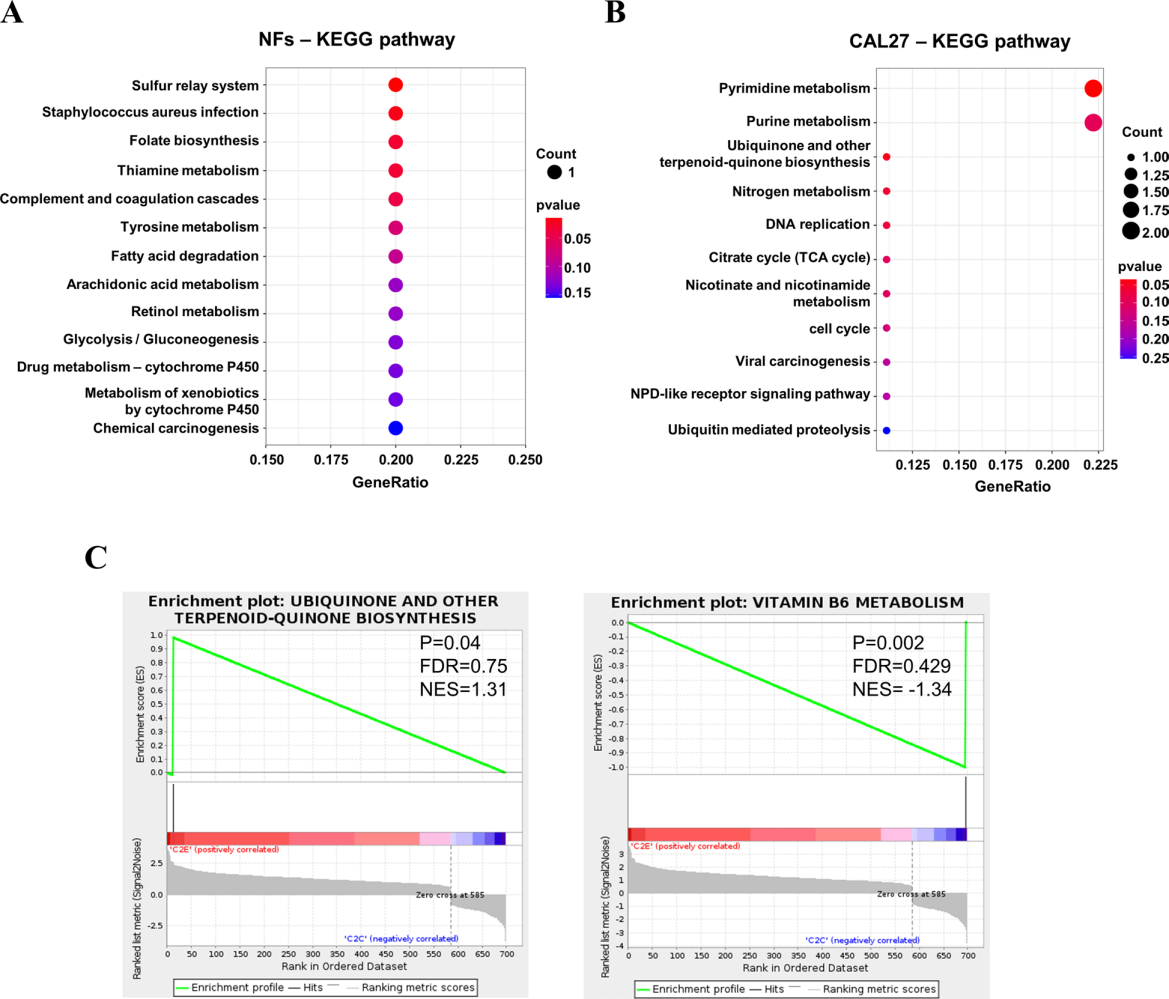
KEGG and GSEA enrichment analysis of DEGs in NFs and CAL27 cells after low-dose arecoline treatment. **A**, **B** For the scatter diagram of KEGG enrichment analysis, KEGG enrichment is measured by gene ratio, p-value, and the number of enriched genes in the pathway. Gene ratio refers to the ratio of the number of DEGs enriched in the pathway to the total number of DEGs. A larger GeneRatio indicates a greater degree of enrichment, and the value range of the p-value is [0,1]. The closer it is to zero, the more significant the enrichment is. **C** GSEA of key pathways in CAL27 cells by induction of low-dose arecoline. *p*-value < 0.05 and normalized enrichment score |NES|> 1 were considered statistically significant. NES, normalized enrichment score. FDR, false discovery rate. Positive and negative NES indicate higher and lower expression after low-dose arecoline treatment. GSEA, gene set enrichment analysis

To further understand the physiological processes after low-dose arecoline treatment, the Kyoto Encyclopedia of Genes and Genomes (KEGG) pathway analysis was performed. The most DEGs enriched pathways in both NFs and CAL27 were metabolism-related pathways. In NFs, low-dose arecoline induced metabolism of some acids and small molecules like thiamine, tyrosine, arachidonic acid and drug metabolism, as well as glycolysis and chemical carcinogenesis (Fig. 3A). In CAL27 cells, pyrimidine metabolism and purine metabolism were significantly enriched pathways, and DNA application and cell cycle were also significantly enriched (Fig. 3B).

Gene Set Enrichment Analysis (GSEA) was performed to determine the contribution of DEGs to the phenotype changes. After low-dose arecoline exposure, 9/13 gene sets were upregulated in NFs, and 15/35 gene sets were upregulated in CAL27 cells. The gene sets with significant enrichment included ubiquinone and another terpenoid-quinone biosynthesis (*p* = 0.04), and vitamin B6 metabolism (*p* = 0.002) (Fig. 3C). These results suggested that low-dose arecoline played roles in fibroblast activation of NFs as well as in DNA application and cell cycle of OSCC cells through regulation of metabolism.

### Identification of protein–protein interaction (PPI) network induced by low-dose arecoline in NFs and OSCC cells

**Fig. 4:**
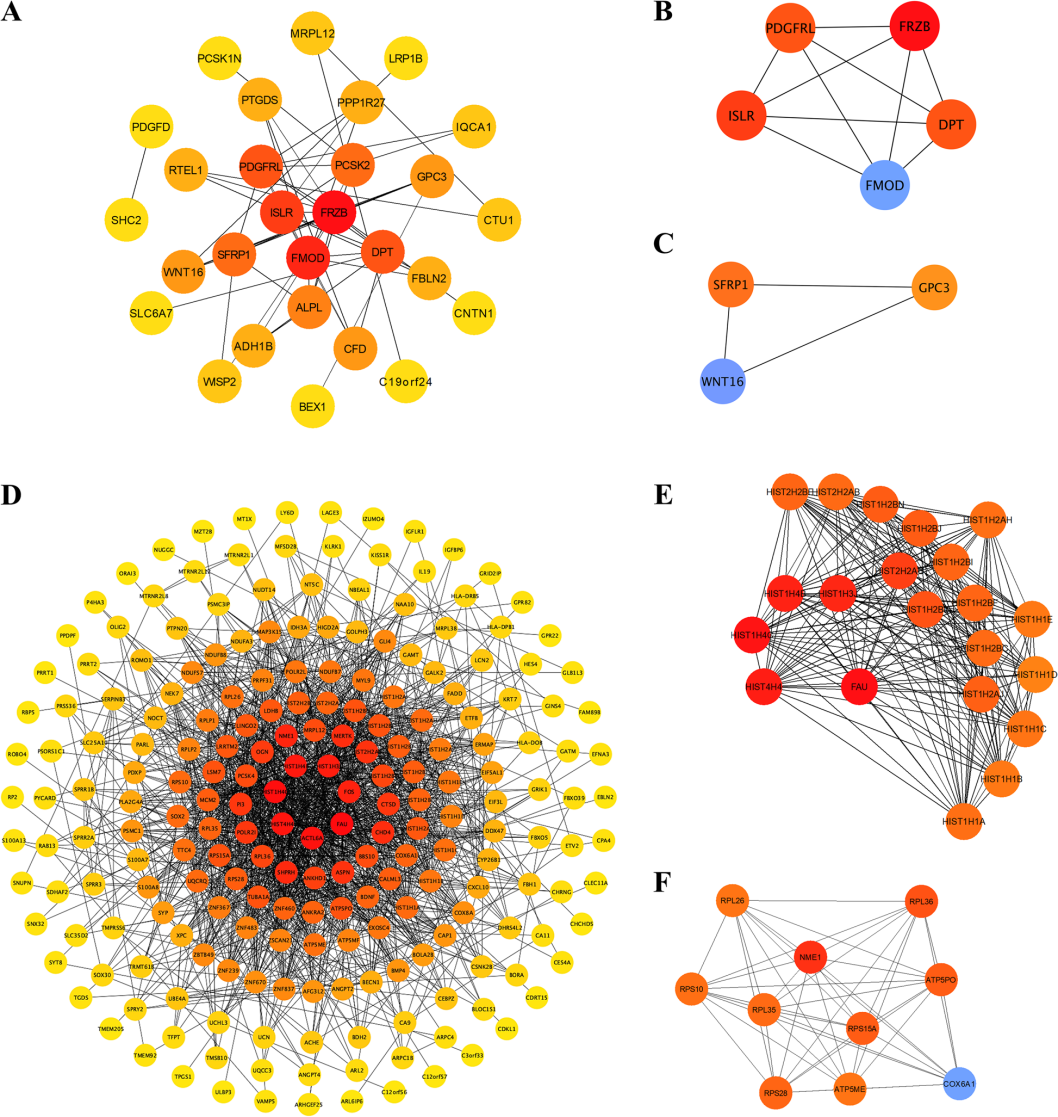
PPI network of DEGs in low-dose arecoline-treated NFs and CAL27 cells. **A** PPI network of arecoline-treated NFs. **B**, **C** The top two most significant modules screened with the Cytoscape plugin MCODE of treated NFs. **D** PPI network of arecoline-treated CAL27 cells. **E**, **F**. The top two most significant modules screened with the Cytoscape plugin MCODE of treated CAL27 cells. The degrees of all nodes were calculated by cytohubba. Color shades were used to indicate degrees. Nodes with a higher degree will have a darker color. C1E, CAL27 cells with low-dose arecoline treatment; C1C, CAL27 cells without low-dose arecoline treatment; NF1E, NFs with low-dose arecoline treatment; NF1C, NFs without low-dose arecoline treatment

To further explore the biological activities of DEGs by low-dose arecoline exposure in NFs and OSCC cells, protein regulatory complexes were constructed via the protein–protein interaction (PPI) network using STRING. For NFs, the network included 218 nodes and 1,585 edges (Fig. 4A), and the top 2 significant modules were identified (Fig. 4B, C). Among them, a Wnt signaling component secreted frizzled‑related protein (FRZB) gene was the most relevant gene, together with another two Wnt signaling regulatory molecules, SFRP1 and WNT16. PPI network also revealed node molecules during low-dose arecoline exposure such as FMOD. FMOD was involved in the assembly of ECM and ISLR/Meflin, a specific marker for mesenchymal stem/stromal cells and fibroblasts. These data suggested that the disruption of Wnt signaling and ECM by low-dose arecoline contributes to OSF pathogenesis.

For CAL27 cells, the network included 218 nodes and 1,585 edges (Fig. 4D). The top 20 significant modules of the network were identified (Fig. 4E, F), among them, the FAU ubiquitin-like and ribosomal protein S30 fusion (FAU) gene was the most relevant gene, followed by H4 clustered histone 3 (HIST1H4C), ACTL6A, and a subunit protein of the BAF (BRG1/brm-associated factor) complex. Moreover, most of the node genes identified were from the histone gene clusters, suggesting low-dose arecoline regulates chromosome assembly during OSCC pathogenesis.

### Low-dose arecoline-induced methylome alterations in NFs and OSCC cells

**Fig. 5:**
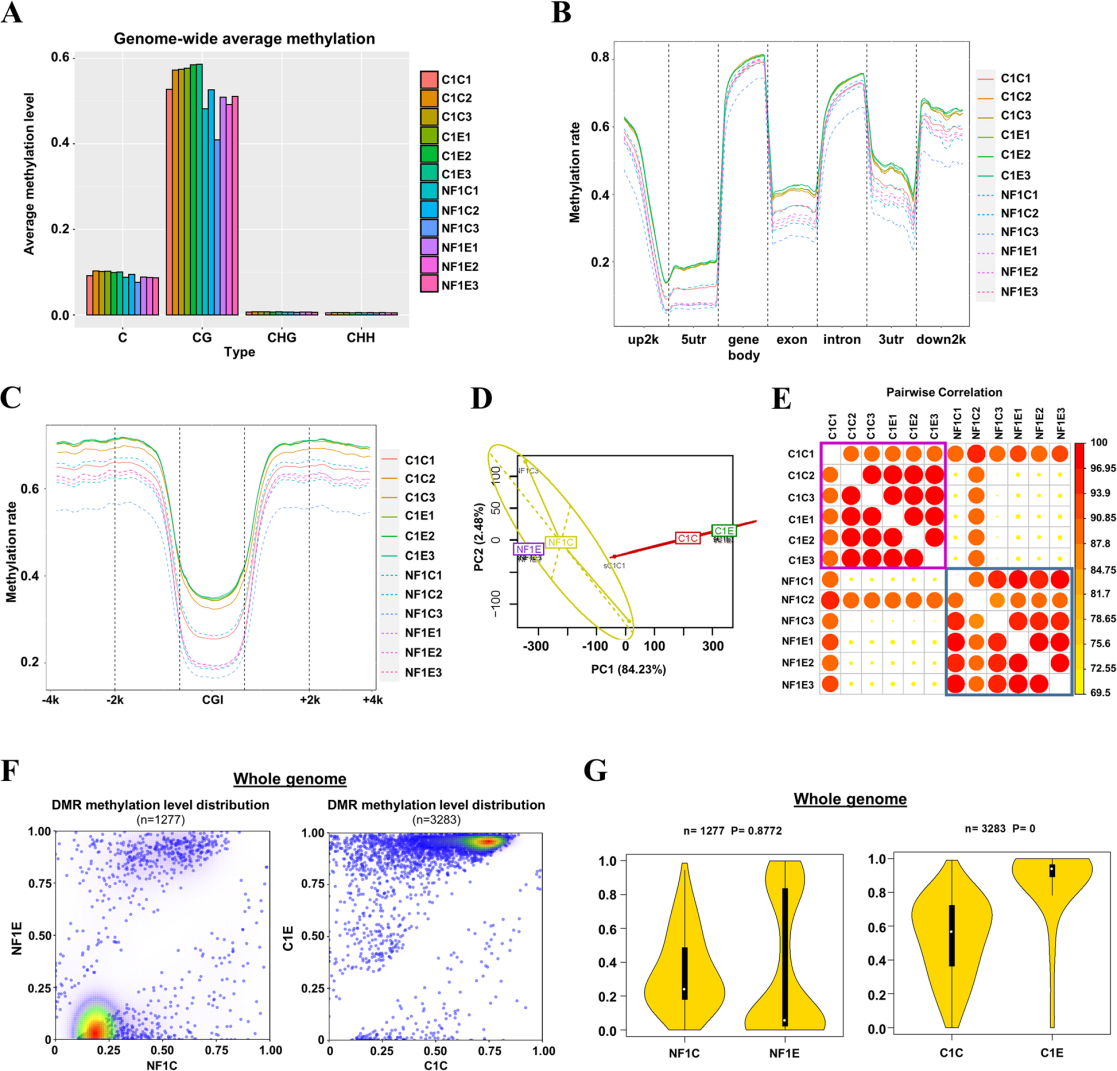
Low-dose arecoline effects on the methylome of NFs and CAL27 cells. **A** Global-wide average methylation of C, CG, CHG and CHH. Bar showed the number of each type of methylated C sites (mCG, mCHG and mCHH) and their proportion in all mC sites. **B** Mean methylation of NFs and CAL27 cells with or without low-dose arecoline treatment levels in different gene elements. **C** Methylation patterns of CGI and its surrounding regions of each sample. **D** Principal component analysis (PCA) for DNA methylation states of 24,949 CpG sites with 16 human cell lines. The PC1 axis clearly distinguishes NFs groups from CAL27 groups, while the PC2 axis distinguishes low-dose arecoline treatment groups from control groups. **E** Pairwise Pearson correlation coefficients (PCC) were calculated for comparison among methylomes of NFs and CAL27 cells with or without low-dose arecoline treatment. Samples were hierarchically clustered with the Euclidean distance method. The color scale indicates the degree of correlation. The correlation matrix and heat map were generated using R software. **F** Pairwise comparison of genome-wide DMR methylation level distribution density between control group and low-dose arecoline treated group in NFs and CAL27s. Density increases from blue to red. DMR density increases from white to red. **G** Violin plot of genome-wide methylation level distribution of DMR in NFs and CAL27s with or without low-dose arecoline treatment. The Y-axis represents the methylation level, and the violin width represents the number of DMRs for that methylation level. The middlebox plot reflects the distribution of DMR methylation levels from another perspective. The average methylation level of DMR between the two groups was calculated using the t-test to calculate the p-value statistics in the plot. C1E, CAL27 cells with low-dose arecoline treatment; C1C, CAL27 cells without low-dose arecoline treatment; NF1E, NFs with low-dose arecoline treatment; NF1C, NFs without low-dose arecoline treatment

As gene expression is regulated by epigenetic mechanisms at transcriptional level, we thus characterized epigenomic changes in NFs and OSCC cells by double-enzyme reduced representation bisulfite sequencing (dRRBS). We firstly evaluated the average methylation level of C bases in all samples, and found methylation levels of CG are higher than those of C, CHG and CHH across the entire transcriptional units (Fig. 5A). We further evaluated methylation alterations of different genetic elements and CpG island (CGI) at different regions (Fig. 5B, C). We observed that methylation rates at gene body, intron, and down2k were hypermethylated, while methylation rates of 5’UTR, exon and 3’UTR were relatively lower (Fig. 5B). We next assessed methylation patterns of CGI and its surrounding regions of each sample, and found the mean methylation levels were higher in CAL27 cells than that of NFs irrelevant to low-dose arecoline treatment. Moreover, after treatment with arecoline, methylation levels of both CAL27 cells and NFs were upregulated compared to control groups (Fig. 5C).

To evaluate methylation differences and similarities in different groups of OSF and OSCC cells, principal component analysis (PCA) and Pearson correlation and cluster (PCC) analysis among samples were performed (Fig. 5D, E). NFs and CAL27 cells with or without low-dose arecoline treatment at each group clustered together on the PCA map, displaying distinct distribution patterns in inner- and inter-groups after low-dose arecoline treatment. Most of the methylome shifts occurred between OSF and OSCC stages, showing the greatest distance from OSF progression stages. Low-dose arecoline exposure caused methylome changes in NFs and CAL27 cells with a relatively short shift (Fig. 5D). Pairwise correlation of gene expression signatures based on Pearson’s correlation coefficients (r) revealed two different types of NFs and CAL27 cells (Fig. 5E).

To identify methylation events in NFs and CAL27 cell genomes after low-dose arecoline exposure, differential methylation region (DMR) numbers in both groups were analyzed. 1,277 DMRs in NFs (Suppl. Table 6) and 3,283 (Suppl. Table 7) DMRs in CAL27 cells were identified under low-dose arecoline exposure (Fig. 5F, G). The density map of DMR methylation level distribution revealed distinct methylation density in NFs and CAL27 cells. CAL27 cells treated with low-dose arecoline reached higher methylation density at whole-genome levels, while the methylation levels of NFs with low-dose arecoline treatment were decreased (Fig. 5F). The violin diagram further confirmed that low-dose arecoline increased methylation levels of CAL27 cells but decreased methylation levels of NFs on average when cells were treated (Fig. 5G). These results suggest that low-dose arecoline-induced methylome alterations in NFs and OSCC cells at different levels.

### Enrichment analysis of DMGs regulated by promoter methylation in NFs and OSCC cells after low-dose arecoline exposure

**Fig. 6:**
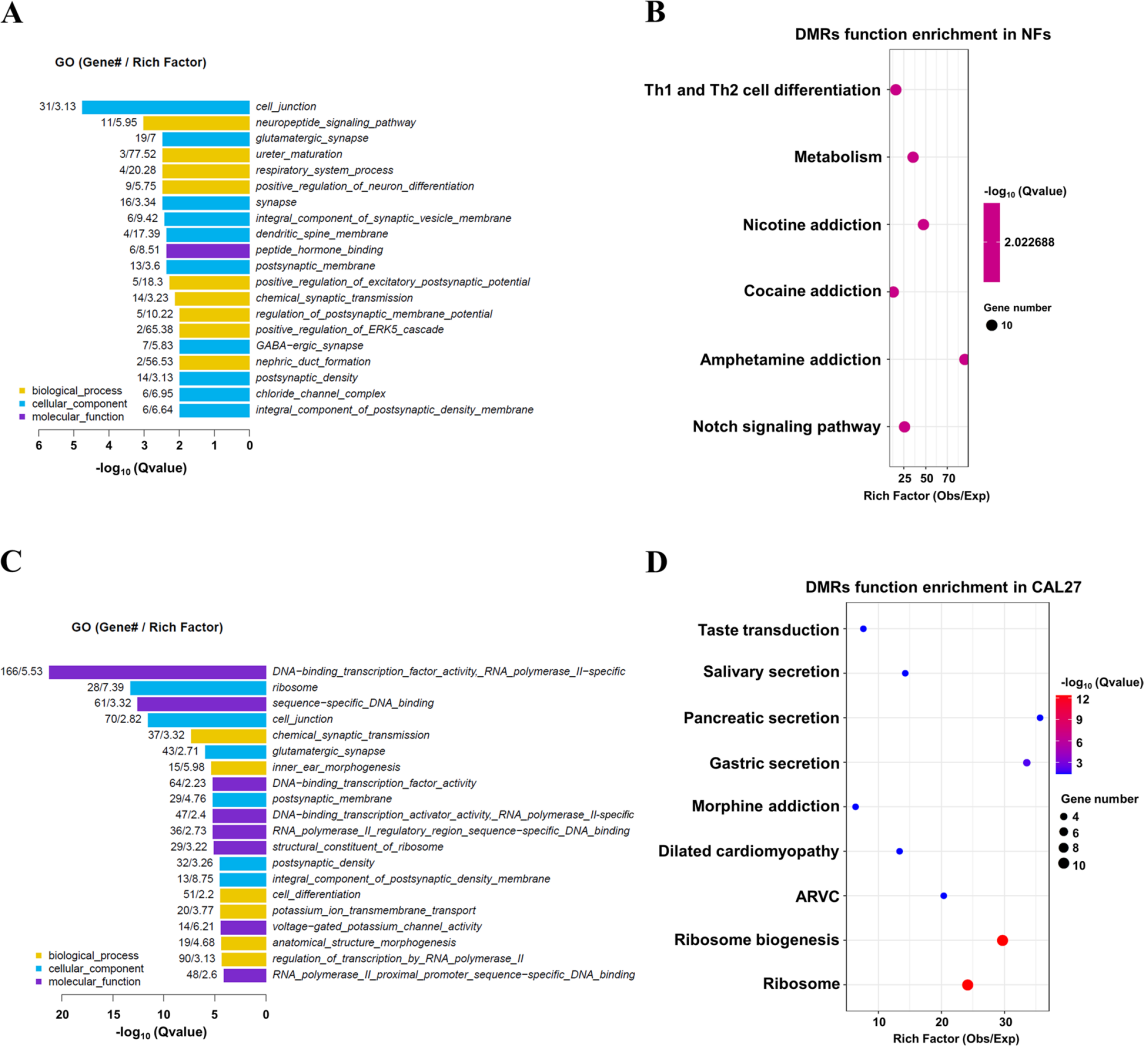
GO and KEGG enrichment analysis of DMRs in the promoter region in NFs and CAL27 cells after low-dose arecoline treatment. Results of GO enrichment analysis of DMRs in the promotor region. GO top20 genes/pathways in NFs **A** and CAL27 cells **C**. Only significant enrichments are shown (Q-value < 0.05). Different color representations differ from the GO three-level classification (biological process, cellular component, and molecular function). The numbers on the left side of the bar represent the number and enrichment multiple of the differential methylated genes enriched into the entry, respectively. The KEGG enrichment of DMRs in NFs **B** and CAL27s **D** after low-dose arecoline treatment. The X-axis represents the enrichment fold. The color of the bubble indicates the size of the q-value, and the size of the bubble indicates the enrichment of the entry total number of genes

As promoter methylation tightly regulates gene transcription, by preventing the binding of RNA polymerases and/or transcriptional factors to the promoter region, we next mainly analyzed the potential functions of differentially expressed genes (DMGs) regulated by promoter methylation in low-dose arecoline-treated NFs and CAL27 cells. After low-dose arecoline exposure, peptide hormone binding was found to be the major molecular function in NFs. Additionally, promoter methylation was enriched in genes that regulate cell junction, synapse, and membrane (Fig. 6A, Suppl. Table 8), involved in Th1 and Th2 cell differentiation, metabolism, chemical addiction (nicotine, cocaine and amphetamine), and the Notch signaling pathway (Fig. 6B). In CAL27 cells, we observed that DNA-binding transcription factor activity was the key function mediated by low-dose arecoline in GO analysis (Fig. 6C, Suppl. Table 9). The most enriched two KEGG pathways were ribosome & ribosome biogenesis, which involved mediating methylome reprogramming of CAL27 cells at the whole-genome level to some extent during OSCC pathogenesis (Fig. 6D).

**Fig. 7:**
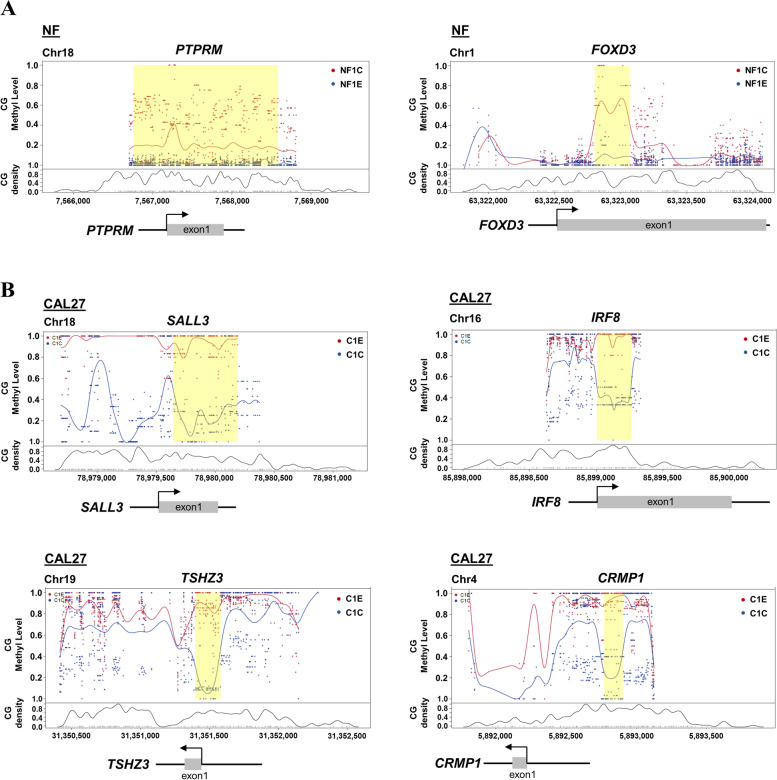
Representative DMG differential methylation curves in the promoter region between control and low-dose arecoline groups. **A** Results of visualization mapping based on q-value screening of differential methylation regions in NFs. **B** Results of visualization mapping based on q-value screening of differential methylation regions in CAL27 cells. q-value < 0.05. The yellow part is the DMR area. The X-axis plots methylation of the 1 kb region extending from the DMR to each side. The top black box indicates the location of the gene promoter and the direction of gene expression in that area. The middle part is the methylation level change curve of the two groups of samples. At the bottom is the CG site density curve. DMG, differentially methylated gene; DMR, differentially methylated region. C1E, CAL27 cells with low-dose arecoline treatment; C1C, CAL27 cells without low-dose arecoline treatment; NF1E, NFs with low-dose arecoline treatment; NF1C, NFs without low-dose arecoline treatment

We then further annotated DMGs between groups after low-dose arecoline treatment by visualization. In the list of top 20 Q-value DMGs in CAL27 cells and NFs, genes with promoter methylation after low-dose arecoline treatment were observed. Low-dose arecoline deceased promotor methylation of *PTPRM* and *FOXD3* in NFs (Fig. 7A). In CAL27 cells, we found hypermethylation of *SALL3, IRF8, TSHZ3* and *CRMP1* at the promotor regions (Fig. 7B). These results suggest that DMGs regulated by promoter methylation play distinct functions during OSF and OSCC pathogenesis after low-dose arecoline exposure.

## Discussion

OSCC is the most common type of head and neck malignancy, and OSF is one of the precancerous lesions of the oral mucosa. Betel nut chewing has been considered a direct causative factor of OSF and OSCC. Studies have shown that arecoline promotes cell proliferation and metastasis [14–16], but most of them are at a single-gene level. The molecular processes during the malignant transformation of OSF and OSCC under arecoline exposure at the whole-genome level remain unknown.

It is well documented that cells respond to arecoline depending on its concentration [17]. Two concentration thresholds have been identified to be biologically relevant: 0.1 µg/ml is critical for collagen stimulation and 10 μg/ml is critical for cytotoxicity [18–23]. Study results related to salivary arecoline levels during chewing of areca nuts revealed that the maximum levels of arecoline occur after chewing for about 25 min due to areca nut particles being deliberately thrown out. The peak level of arecoline varies greatly among different individuals [24, 25]. Moreover, arecoline concentrations are above the 0.1 μg/ml level at 50 min in most participants. However, the data about arecoline concentration after chewing 24 h or later is still unclear, although salivary arecoline levels of many betel chewers are often above the 0.1 μg/ml threshold, suggesting that low-dose arecoline could be maintained in the oral cavity of betel nut chewers with long-term and high-frequency chewing. Thus, there is a possibility that heavy betel quid chewers are at a higher risk of developing OSF due to a prolonged and low-dose arecoline microenvironment found in the oral cavity, whereas chewing areca nut produces high-dose arecoline for only a short period of time.

In this study, we investigated the effects of arecoline in different dosages on cell proliferation and cell cycle. We found that arecoline has a dose-dependent effect on both fibroblast and oral squamous epithelial cells. A high dose is cytotoxic to both NFs and CAL27 cells, while a low dose can promote cell proliferation and accelerate cell cycle progression, consistent with previous studies [16, 26, 27]. In our pilot data, we found that after 48 h of arecoline treatment with different low-dose concentrations, there was a very small difference between the groups. Thus, in this study, we selected low-dose arecoline and 24-h treatment to mimic the in vivo low-dose oral microenvironment in betel nut chewers. It is noted that low doses of arecoline are more reflective of daily doses of betel nut chewing. Thus, we chose low-dose arecoline for our following mechanistic studies.

Studies investigated changes in morphology and responses to various concentrations of arecoline by culturing human oral fibroblasts from the normal buccal mucosa and OSF tissues. Results showed that both NF and OSF fibroblasts have similar doubling times and responses to various arecoline concentrations, as well as could reach the same level of collagen synthesis rate after arecoline treatments, except that NFs have slightly higher numbers of cells due to zero exposure to arecoline before, and OSF fibroblasts have significantly higher the ratio of F3 (stellate-shaped) to F1 (spindle-shaped) cells [28, 29]. Epigenetic changes are considered as the earliest and most comprehensive genomic aberrations occurring during tumor pathogenesis, facilitating tumor initiation and progression [30]. To assess the early effects of genome-wide methylation changes during OSF pathogenesis under low-dose arecoline exposure, we opted to use NF as one of the cell models in this study.

DNA methylation can be influenced by environmental factors including exposure to environmental chemicals [31], suggesting that arecoline could contribute to OSCC malignant progression. In this study, we treated CAL27 with low-dose arecoline and observed genome-wide increased methylation levels and changes in the gene expression profile of CAL27 cells. However, using betel-associated OSCC cell lines or NHOK could be more appropriate as epithelial counterparts, such as the OC3 cell line, established from an areca nut chewing/non-smoker OSCC patient with long-term areca (betel) chewing and non-smoke, which possesses both features of keratinocyte and neoplasm [32]. Further investigation of the effects of arecoline on normal human oral keratinocytes, OSF fibroblasts and betel-associated OSCC cells needs to be conducted.

We next performed RNA-Seq to examine the transcriptome alterations of NFs and CAL27 under low-dose arecoline exposure. We found 92 DEGs in NFs and 698 DEGs in CAL27 cells. Some of the DEGs have previously been reported associated with OSF and/or OSCC, like *S100A7* [33–36], *RPS28* [37] and *CYP26B1* [38, 39]. Increased expression of CYP26B1 was identified in betel quid (BQ)-related OSCC tumor tissues, and could be a novel biomarker for BQ-related oral and pharyngeal cancers [38, 39]. In our study, we also observed *CYP26B1* upregulation in CAL27 cells after low-dose arecoline treatment, which further supports its novel role in the BQ-dependent pathogenesis of OSCC. GO analysis showed that low-dose arecoline treatment caused changes in the extracellular matrix in NFs, and was involved in chromatin organization in CAL27 cells, indicating that low-dose arecoline exposure plays distinct roles by regulating DEGs during OSF and OSCC pathogenesis. In the OSF stage, low-dose arecoline regulates ECM components and is further involved in fibroblast survival, migration, and metabolism. In NFs, the significantly upregulated gene is fibromodulin (FMOD), an important ECM small leucine-rich proteoglycans protein, which plays a critical role in ECM organization and cancer pathogenesis [40]. FMOD led to liver and lung fibrosis by promoting collagen I deposition [41]. FMOD promoted angiogenesis by downregulating multiple microenvironmental factors including VEGF, TGF-β1, FGF-2, and PDGF-B [42, 43]. FMOD upregulation in NFs after low-dose arecoline treatment indicated that arecoline could improve the fiber-forming ability of fibroblasts during OSF pathogenesis.

Activation of the Wnt signaling pathway has a crucial role in regulating fibroblast activation and collagen release in fibrosis, further inducing fibrosis [44, 45]. We observed Wnt protein binding (SFRP1, SFRP3/FRZB) and frizzled binding (WNT16, SFRP1) involved in arecoline-induced OSF pathogenesis in our study. High *SFRP1* and *SFRP3* expression in NFs after low-dose arecoline treatment may facilitate stromal-epithelial cell signaling and be involved in remodeling the epithelial compartment. Further investigation is needed to elucidate the molecular mechanisms of Wnt-related proteins in regulating stromal-epithelial interaction and ECM remodeling during OSF pathogenesis.

Distinct roles of enriched DEGs induced by low-dose arecoline were found in CAL27 cells, which mainly regulated protein metabolic processes and chromatin organization. PPI assay also showed histone gene clusters as node genes during arecoline-triggered OSCC pathogenesis. We thus further analyzed methylome changes induced by low-dose arecoline during OSF and OSCC pathogenesis using the dRRBS platform. As expected, the genome-wide methylation level was increased in CAL27 cells after low-dose arecoline induction but decreased in NFs with hypermethylation in specific gene loci, consistent with DEGs regulation by RNA-seq.

Aberrant promoter methylation has been well-recognized as a hallmark of cancer, through silencing tumor suppressor genes and activating oncogenes [46, 47]. We then focused on DMRs regulated by promoter methylation under low-dose arecoline exposure. Further GO and KEGG analysis revealed disruption of Th1 and Th2 cell differentiation, metabolism, chemical addiction (nicotine, cocaine and amphetamine), and the Notch signaling pathway during arecoline-induced OSF pathogenesis. Interestingly, low-dose arecoline promotes dependence on nicotine, cocaine, and amphetamine, which explains the synergistic effect of betel nut chewing, smoking, and drinking on OSF malignancy. Significant DMRs in CAL27 cells were enriched in ribosome biogenesis and ribosome, supporting the key role of low-dose arecoline in chromatin reorganization and further methylome reprogramming during OSCC pathogenesis.

At the early stages of tumorigenesis, promoter methylation of tumor suppressor genes is often observed at varying frequencies, suggesting its potential as a biomarker and regulator of transcription [48, 49]. Studies have shown that epigenetic dysregulation of transcription factor *FOXD3 *by helicobacter pylori promoted gastric carcinogenesis, indicating *FOXD3* methylation during the early stage of gastric tumorigenesis [50]. FOXD3 as an epigenetic regulator mediates the transcriptional repression of multiple cancer genes including *SLC25A26* and *NANOG* [51, 52]. The protein tyrosine phosphatase receptor-like gene *PTPRM* was frequently methylated in sporadic colorectal cancer [53]. Loss of *PTPTM* by genetic and epigenetic alterations in colon adenomas and carcinomas contributed to the early step of colorectal tumorigenesis [54]. Here, we observed the promoter methylation of *PTPRM* and *FOXD3* after low-dose arecoline treatment in NFs, indicating the roles of *PTPRM* and *FOXD3* methylation regulation in oral fibroblast activation and fibrogenesis under low-dose arecoline treatment. Thus, *PTPRM* and *FOXD3* demethylation could be the early event during OSF pathogenesis and potential biomarkers for OSF progression.

In CAL27 cells induced by low-dose arecoline, we observed promoter methylation of *SALL3, IRF8, TSHZ3* and *CRMP1*. *SALL3* methylation could clearly distinguish HNSCC from adjacent normal mucosal tissue, thus being an independent predictor of poor survival in HNSCC [55]. Significantly higher *IRF8* promoter methylation has been identified in multiple carcinomas. The MDSC-IL-10-STAT3-DNMT3b-IRF8 pathway is also involved in the molecular progression from inflammation to colon cancer initiation [56]. *TSHZ3* promoter methylation was found in breast and prostate cancer cells [57]. *CRMP1* methylation was highest in HCC tissues, and higher in cirrhotic liver tissues than in normal live tissues. This indicates its methylation during the progression of the cirrhotic liver to HCC [58]. Our findings in CAL27 cells indicated the critical role of methylation of *SALL3, IRF8, TSHZ3* and *CRMP1* in the OSCC progression under low-dose arecoline exposure. Our results also confirmed that promoter methylation of *RARB* is upregulated in low-dose arecoline-treated CAL27 cells, consistent with the finding of *RARB *hypermethylation in areca-associated oral carcinogenesis [10].

Integrating transcriptome analysis and DNA methylome provided a direct correlation between DNA methylation and gene transcription in NFs and CAL27 cells, which are complementary to each other to reveal gene expression profiling and distinct biological functions induced by low-dose arecoline exposure during OSF and OSCC pathogenesis. Besides different roles in OSF and OSCC stages, low-dose arecoline regulated metabolic pathways in both cells at different levels. In NFs, disorders of folate biosynthesis and thiamine metabolism could lead to impaired metabolism of glucose and amino acids, further progression of fibrosis. Recent studies have demonstrated that disruption of nucleotide metabolism not only accelerates cancer development but also defects normal immune response in the tumor microenvironment [59]. In CAL27 cells, we found that low-dose arecoline was involved in ubiquinone and another terpenoid-quinone biosynthesis and nucleotide metabolism, indicating the roles of low-dose arecoline in OSCC development and tumor microenvironment. Further investigation of the effects of low-dose arecoline in the tumor microenvironment of OSCC is needed.

In conclusion, we have performed a comprehensive analysis of the transcriptome and methylome of low-dose arecoline in OSF and OSCC cells, exploring for the first time the contribution of arecoline-induced epigenetic reprogramming to molecular pathogenesis in OSF and OSCC. Two “omics” levels of analyses revealed distinct functions mediated by low-dose arecoline during OSF and OSCC pathogenesis. Some key candidate genes and key signaling pathways that participate in metabolism disorders have been identified, specializing in ECM modulation in OSF cells and chromatin organization in OSCC cells. Elucidation of early molecular events during OSF and OSCC pathogenesis could serve as potential biomarkers for early diagnosis and prognosis for OSF and OSCC. Future studies uncovering the biological functions of the identified genes above will further our understanding of the carcinogenesis of arecoline and provide new sites for the clinical treatment of OSF and OSCC.

## Supplementary Information

**Additional file 1:** **Suppl. Table 1.** Summary of DEGs in NFs samples with or without low-dose arecoline treatment.

**Additional file 2:** **Suppl. Table 2.** Summary of DEGs in CAL27 samples with or without low-dose arecoline treatment.

**Additional file 3:** **Suppl. Table 3.** 10 common DEGs in NFs and CAL27 cells after low-dose arecoline treatment.

**Additional file 4:** **Suppl. Table 4.** Summary of GO enrichment in NFs under low-dose arecoline treatment comparedwith control group. 

**Additional file 5:** **Suppl. Table 5.** Summary of GO enrichment in CAL27 cells under low-dose arecolinetreatment compared with control group. 

**Additional file 6:** **Suppl. Table 6.** Summary of DMRs in NFs samples with or without low-dose arecoline treatment.

**Additional file 7:** **Suppl. Table 7.** Summary of DMRs in CAL27 samples with or without low-dose arecoline treatment.

**Additional file 8:** **Suppl. Table 8.** Summary of DMGs at promoter and gene body in NFs samples with or without low-dose arecolinetreatment.

**Additional file 9:** **Suppl. Table 9.** Summary of DMGs at promoter and gene body in CAL27 samples with or without low-dose arecolinetreatment.

## Data Availability

The datasets generated and analysed during the current study are available in the NCBI under the accession number PRJNA911851.
